# Anti-SEZ6L2 antibodies in paraneoplastic cerebellar syndrome: case report and review of the literature

**DOI:** 10.1186/s42466-022-00218-4

**Published:** 2022-10-31

**Authors:** Annika Kather, Florian Holtbernd, Robert Brunkhorst, Dimah Hasan, Robert Markewitz, Klaus-Peter Wandinger, Martin Wiesmann, Jörg B. Schulz, Simone C. Tauber

**Affiliations:** 1 Department of Neurology, University Hospital RWTH Aachen, Aachen, Germany; 2 Department of Diagnostic and Interventional Neuroradiology, University Hospital RWTH Aachen, Aachen, Germany; 3 Institute of Clinical Chemistry and Department of Neurology, University Hospital Schleswig-Holstein, Lübeck, Germany

## Abstract

Seizure Related 6 Homolog Like 2 (SEZ6L2) protein has been shown to have implications in neuronal and especially motor function development. In oncology, overexpression of SEZ6L2 serves as a negative prognostic marker in several tumor entities. Recently, few cases of anti-SEZ6L2 antibody mediated cerebellar syndromes were reported. In this article, we present a case of a 70-year-old woman with subacute onset of gait disturbance, dysarthria and limb ataxia. Serum anti-SEZ6L2 antibodies were markedly increased, and further diagnostic workup revealed left sided breast cancer. Neurological symptoms and SEZ6L2 titer significantly improved after curative tumor therapy. This is a very rare and educationally important report of anti-SEZ6L2 autoimmune cerebellar syndrome with a paraneoplastic etiology. Additionally, we performed a review of the current literature for SEZ6L2, focusing on comparing the published cases on autoimmune cerebellar syndrome.

## Background

In recent years, several antibodies linked to specific clinical neurological syndromes have been identified [1], indicating the need of a thorough testing for these antibodies in cases with unexplained neurological deficits. This is especially of relevance as these antibody-mediated diseases are treatable. In well-known entities such as anti-NMDAR-encephalitis, the underlying mechanisms are well understood and treatment responses are often favorable [2, 3]. For many other antibody-mediated autoimmune disorders, knowledge regarding origin and treatment options is still lacking.

The Seizure Related 6 (SEZ6) protein family came into the focus of epilepsy research in the 1990s [4]. In the brain, the cell surface protein Seizure Related 6 Homolog Like 2 (SEZ6L2) is a part of the α-Amino-3-hydroxy-5-methyl-4-isoxazolepropionic acid (AMPA) receptor, and is highly expressed in the cerebellar cortex, contributing to neuronal growth [5, 6]. Furthermore, in its function as receptor of cathepsin D, SEZ6L2 is a mediator of motor function development [7]. In autism spectrum disorders, mutations in the SEZ6L2 gene were identified [8]. Further research also linked the gene to other psychiatric diseases [9].

Apart from neurosciences, SEZ6L2-expression has also been linked to different types of cancer [10, 11], is associated with poor outcome [12, 13], and therefore can serve as a biomarker [14] and possible therapeutic target [15].

Anti-SEZ6L2 autoimmune cerebellar syndrome was first reported in 2014 [16]. Only a few case reports are available with variable treatment responses. Here, we present the first paraneoplastic case of anti-SEZ6L2 autoimmune cerebellar syndrome caused by breast cancer.

## Case report

**Fig. 1:**
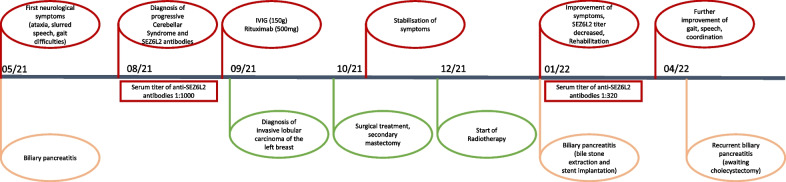
Course of the disease in chronological order

A 70-year-old female presented with slurred speech, ataxia and abnormal gait resulting in multiple falls. Symptoms were progressive and first presented three months prior to admission (Fig. 1). Around that time, the patient suffered from a biliary pancreatitis. Her medical history also included hypertonia treated with beta-blockers and a recently diagnosed depression treated with a selective serotonin reuptake inhibitor (SSRI). There was no family history of neurological disorders.

On examination, we found saccadic eye movements and impaired vestibulo-ocular reflex suppression, scanning speech and dysarthria, right sided limb hemiataxia, increased reflexes in the left arm and right leg with ankle clonus and positive Babinski sign. Gait was profoundly impaired by ataxia.

Blood analysis did not indicate a metabolic cause of the cerebellar syndrome. Cerebrospinal fluid (CSF) analysis resulted in normal cell count and protein levels. Oligoclonal bands (OCB) were positive. Phospho-tau was slightly increased to 71 pg/ml (normal range < 61). All other parameters including cytology were normal. However, auto-antibody panel analysis for autoimmune encephalitis/cerebellitis was positive for anti-SEZ6L2 antibodies with a titer of 1:1000 in the serum (reference range: < 1:10). Anti-SEZ6L2 antibodies were detected by immunohistochemistry on cerebellar tissue and were confirmed by specifically transfected human embryonic kidney (HEK) cells. CSF was not tested for anti-SEZ6L2 antibodies due to lack of material.

**Fig. 2:**
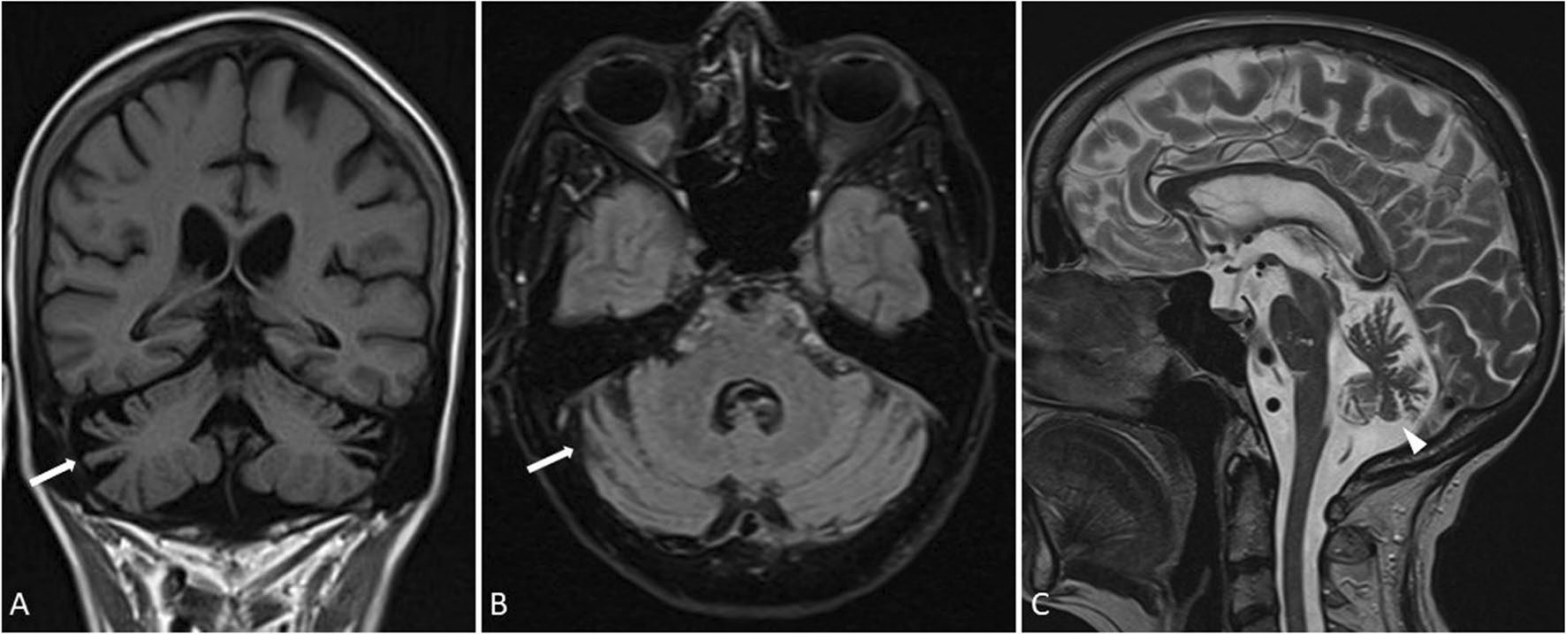
MR image of the patient shows atrophy of cerebellar hemispheres (arrows) in Axial T2-FLAIR images **a** and coronal T1-weighted images **b** and vermis atrophy (arrowhead) in sagittal T2-weighted images (**c**)

Brain MRI showed atrophy of the vermis and cerebellar hemispheres (Fig. 2). Spinal MRI revealed no pathologies of the spinal cord.

The patient was started on a five-day course of intravenous immunoglobulins (IVIG, total dose 150 g) followed by a single cycle of rituximab (500 mg).

Whole body 18F FDG-PET-CT suspected breast cancer. Further work up revealed an invasive lobular carcinoma of the left breast (Fig. 1). The patient was referred to gynecology. Immunohistochemistry revealed estrogene receptor positivity (100%), progesterone receptor positivity (15%), and HER2-neu negativity. After mastectomy including sentinel lymph node excision, adjuvant radiotherapy was performed, followed by aromatase-inhibitor therapy.

At this stage of her treatment, we followed up on the patient. She reported stabilization of speech and gait without further deterioration, this was congruent with the neurological examination. We decided against further cycles of rituximab due to improvement of symptoms.

Rehabilitation was delayed because the patient suffered from another biliary pancreatitis (Fig. 1), treated by bile duct stone extraction and stent implantation. At this point, neurological examination revealed a less severe gait and only slight limb ataxia and the patient reported profound stabilization of gait using a walker as well as improvement of speech fluency and articulation (Fig. 1). Anti-SEZ6L2 antibody titer in the serum decreased to 1:320.

Three month later, after rehabilitation, the patient presented to our outpatient clinic, reporting profound improvement regarding walking distance, speed and coordination and nearly normalization of speech. Neurological examination still showed saccadic eye movements and a left sided ataxia in the arm and leg. Gait was more fluent and secure, although still clearly impaired (Fig. 1). Cholecystectomy was planned due to recurrent pancreatitis.

## Published cases of SEZ6L2 associated cerebellar syndrome

**Fig. 3:**
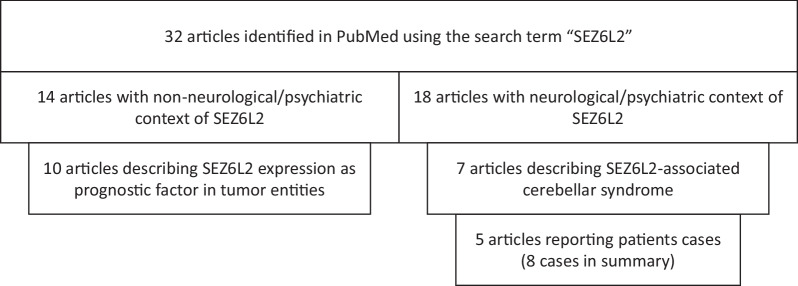
Review of published literature on SEZ6L2

Up to now, eight cases of SEZ6L2-associated cerebellar syndrome have been reported in the literature (Fig. 3, Table 1). It was first described 2014 in a 60-year-old female patient who presented with additional retinopathy [16]. Cerebellar syndrome was associated with parkinsonism in five of the eight patients [17]. Cognitive dysfunction was reported in two cases [18, 19]. All but two reported cases had normal standard CSF parameters. In one case, a pleocytosis was found [18], in another case high protein levels [19]. In two cases OCB were examined, with a negative result [17, 18]. Positive OCB, as observed in our patient, were not reported. Abnormalities of neurodegenerative markers were reported in two cases [17, 19]. Brain MRI of a few of the cases showed cerebellar atrophy. One case with predominantly cognitive function abnormalities presented with hippocampal atrophy [19]. An underlying malignancy proposing a paraneoplastic mechanism was found in a patient years after onset of cerebellar symptoms [18]. In another report, small cell lung cancer (SCLC) was diagnosed after onset of neurological symptoms [19]. Immunosuppressive therapy was applied in all patients. The outcome was mostly unfavorable. A positive outcome was only reported in two of the eight cases, one after receiving cyclophosphamide [20], the other after receiving an immunotherapy not further specified in the paper [16]. The patient with SCLC received immunosuppressive therapy as well as cancer treatment and died after five months. Our patient improved after breast cancer treatment, preceded by IVIG treatment and a single dose of Rituximab.

**Table 1:** Review of published cases of SEZ6L2 associated cerebellar syndrome

References	Cases (comorbidities if specified)	Symptoms	Diagnostic findings	Treatment	Outcome
Yaguchi et al. [16]	60yo F	Severe ataxia, retinopathy	CSF: normal MRI brain: normal (cerebellar atrophy after 2 y) No malignancy found	Immunotherapy (no further specification given)	Mild improvement (24 months follow up)
Borsche et al. [17]	55yo F with Crohn’s disease	Square wave jerks Limb Ataxia Impaired gait Postural instability	CSF: normal cell count and protein levels, OCB negative, beta-amyloid levels decreased, normal tau levels MRI brain: cerebellar atrophy PET-CT: normal	IVIG Rituximab	Deterioration in spite of IVIG, improvement after Rituximab (12 month follow up)
Landa et al. [18]	69yo M	Dysarthria, gait ataxia, headache, postural instability, apraxia, echolalia, axial rigidity, hypomimia, bradykinesia, hypophonia, diplopia, saccadic eye movements	CSF: Pleocytosis (90/µl), normal glucose and protein levels, OCB negative MRI brain: normal PET-CT: normal	IVMP IVIG Rituximab Cyclophosphamide	Further deterioration (10 months follow up)
	55yo F	Dysarthria, gait ataxia, limb ataxia, cognitive impairment, unilateral parkinsonism, downbeating and torsional nystagmus	CSF: normal MRI brain: normal Metastatic ovarian cancer (4 years after onset auf cerebellar syndrome)	IVMP IVIG	Further deterioration (death after 52 months from ovarian cancer)
	54yo M	Dysarthria, gait ataxia, limb ataxia, mild cognitive impairment, bradykinesia, saccadic eye movements, end-gaze nystagmus	CSF: normal MRI brain: normal No malignancy found	IVMP Plasmapheresis	Further deterioration (36 months follow up)
	69yo F	Dysarthria, gait ataxia, limb ataxia, downbeat nystagmus	CSF: normal MRI brain: normal No malignancy found	Prednisone Cyclophosphamide	Further deterioration (72 months follow up)
Mehdiyeva et al. [20]	73yo F with depression	Nausea Bilateral gaze-evoked nystagmus, dysarthria, truncal ataxia, Postural instability, hypophonia, bradykinesia	MRI brain: cerebellar atrophy PET-CT: normal	IVMP IVIG Rituximab Cyclophosphamide	Marked improvement with cyclophosphamide (15 months follow up)
Carneiro et al. [19]	62yo F with mild hyponatremia, hypothyroidism	Gait ataxia, limb ataxia Disorientation, anterograde memory loss	CSF: high protein (87 mg/dL), high tau (2130 pg/mL, reference < 335 pg/mL) MRI: hippocampal atrophy, T2-hyperintensity of right hippocampus PET-CT: lung carcinoma suspected, confirmed by further evaluation	IVMP IVIG Cancer treatment	Death after 5 months
Our case 2022	70yo F with recurrent biliary pancreatitis, depression	saccadic eye movements, disrupted vestibulo-ocular reflex, scanning speech and dysarthria, right sided limb hemiataxia, increased reflexes in the left arm and right leg with ankle clonus and pyramidal signs, profound gait ataxia	CSF: normal cell count and protein levels, OCB positive, phospho-tau increased (71 pg/ml, normal range < 61) MRI brain: cerebellar atrophy PET-CT: Mamma Ca suspected, confirmed by further evaluation	IVIG Rituximab (once) Cancer treatment	Improvement after mastectomy (8 months follow up)

*yo* years old, *F* female, *M* male, *CSF* cerebrospinal fluid, *MRI* magnet resonance imaging, *OCB* oligoclonal bands, *PET-CT* positron emission tomography computer tomography, *IVIG* intravenous immunoglobulins, *IVMP* intravenous methylprednisolone

## SEZ6L2 in other clinical contexts

Apart from autoimmune cerebellar syndrome, SEZ6L2 also plays a role in other clinical contexts in neurology and psychiatry (Fig. 3).

In an animal study using knock-out mice, SEZ6L2 was found to have an influence on motor skill and coordination development [21]. SEZ6L2 has been proposed as a CSF biomarker differentiating idiopathic normal pressure hydrocephalus from Alzheimer’s disease [22]. High gene expression levels of SEZ6L2 in patients with glioblastoma were found to be a negative prognostic factor [12]. In degenerative disc disease, high gene expression of SEZ6L2 has been associated with an inflammatory etiology [23]. Mutation in the SEZ6L2 gene and the broader family of SEZ6 proteins are also in the focus of research on autism [24], febrile seizures in children [25, 26], bipolar disorder [9] and schizophrenia [27].

Outside the neurological and psychiatric field, high SEZ6L2 gene expression in tumor tissue is a negative prognostic factor in various tumor entities. Specifically, overexpression of SEZ6L2 has been linked to an unfavorable outcome in glioblastoma [12], colorectal cancer [28, 29], cholangiocarcinoma [13], lung adenocarcinoma [15], non-small cell lung cancer [10], thyroid cancer [30], hepatocellular carcinoma [31], and breast cancer [11]. In ovarian cancer, SEZ6L2 was shown to be a serological biomarker [14]. For osteosarcoma, an upregulation of SEZ6L2 was associated with methylation [32].

Interestingly, in a mouse model of lung adenocarcinoma, anti-SEZ6L2 antibodies had a positive effect on drug resistance and metastasis [15].

Apart from being upregulated in malignancies, SEZ6 proteins play a role in complement regulation [33] and in the pancreas, SEZ6L2 is specific to islet cells [34].

## Discussion

With our case report, we add knowledge to the newly discovered entity of anti-SEZ6L2 mediated autoimmune cerebellar syndrome. Uniquely, we detected a paraneoplastic origin of this entity due to breast cancer. Symptoms markedly improved after curative cancer therapy. Interestingly, our patient suffered from biliary pancreatitis recurrently during the cerebellar syndrome. As SEZ6L2 is a marker of pancreatic islet cells [34], a link to a predisposition to pancreatitis with increased anti-SEZ6L2 antibodies in the body could be speculated.

There is evidence for direct pathogenicity of the anti-SEZ6L2 antibodies in the development of cerebellar syndrome [5]. Nonetheless, considering the various roles SEZ6L2 plays as part of the AMPA receptor [6] and in cathepsin D transport [7], a degenerative mechanism could also be involved. Further research is needed to address this question.

The paraneoplastic origin of the cerebellar syndrome observed in our patient is especially interesting in the light of the prognostic value of SEZ6L2 expression in various types of cancer [10–15, 28–31]. In the future, anti-SEZ6L2 treatment might be possible to positively influence the course of malignancies [15]. As neurologists experienced with side effects of checkpoint inhibitors [35], this approach could also trigger autoimmune side effects resulting in encephalitis or cerebellitis. Therefore, SEZ6L2 antibody mediated autoimmune cerebellar syndrome should be on the list of differential diagnosis for subacute ataxia now and in the future.

## Data Availability

Data sharing is not applicable to this article as no datasets were generated or analysed during the current study.
